# Whole Genome Sequencing Identifies Novel Compound Heterozygous Lysosomal Trafficking Regulator Gene Mutations Associated with Autosomal Recessive Chediak-Higashi Syndrome

**DOI:** 10.1038/srep41308

**Published:** 2017-02-01

**Authors:** Yaqiong Jin, Li Zhang, Senfen Wang, Feng Chen, Yang Gu, Enyu Hong, Yongbo Yu, Xin Ni, Yongli Guo, Tieliu Shi, Zigang Xu

**Affiliations:** 1 Beijing Key Laboratory for Pediatric Diseases of Otolaryngology, Head and Neck Surgery, MOE Key Laboratory of Major Diseases in Children, Beijing Pediatric Research Institute, Beijing Children’s Hospital, Capital Medical University, Beijing, China; 2 Biobank for Clinical Data and Samples in Pediatric, Beijing Pediatric Research Institute, Beijing Children’s Hospital, Capital Medical University, Beijing, China; 3 Center for Bioinformatics and Computational Biology, and the Institute of Biomedical Sciences, School of Life Sciences, East China Normal University, Shanghai, China; 4 Department of Dermatology, Beijing Children’s Hospital, Capital Medical University, Beijing, China; 5 Department of Otolaryngology, Head and Neck Surgery, Beijing Children’s Hospital, Capital Medical University, Beijing, China

## Abstract

Chediak–Higashi syndrome (CHS) is a rare autosomal recessive disease characterized by varying degrees of oculocutaneous albinism, recurrent infections, and a mild bleeding tendency, with late neurologic dysfunction. This syndrome is molecularly characterized by pathognomonic mutations in the *LYST* (lysosomal trafficking regulator). Using whole genome sequencing (WGS) we attempted to identify novel mutations of CHS based on a family of CHS with atypical symptoms. The two patients demonstrated a phenotypic constellation including partial oculocutaneous albinism, frequency upper respiratory infection or a marginal intelligence, without bleeding tendency and severe immunodeficiency. WGS revealed two compound *LYST* mutations including a maternally inherited chr1:235969126G > A (rs80338652) and a novel paternally inherited chr1: 235915327A > AT, associated with autosomal recessive CHS. These two variants fall in the coding regions of *LYST*, resulting in premature truncation of LYST due to R1104X/N2535KfsX2 induced incomplete translation. Notably, the heterozygous carriers (i.e. parents) were unaffected. Our finding also reveals decreased plasma serotonin levels in patients with CHS compared with unaffected individuals for the first time. The present study contributes to improved understanding of the causes of this disease and provides new ideas for possible treatments.

Chediak-Higashi Syndrome (CHS) is a rare autosomal recessive disease, characterized by partial oculocutaneous albinism (OCA), increased susceptibility to infection, a mild bleeding tendency, and/or late-onset progressive neurological impairment^1^. Bone marrow transplantation (BMT) is an acceptable curative treatment for CHS^2^. Defective structure or function of melanosomes in melanocytes is causative of OCA in CHS^3^. Large eosinophilic, peroxidase-positive inclusion bodies in circulating granulocytes cytotoxic T lymphocytes (CTLs), NK cells, and macrophages increase susceptibility to infection of patients with CHS^4^. The absence of dense bodies in CHS result in mild bleeding tendencies due to the inability to form platelet aggregation and hemostatic plugs^1^,^3^,^5^. Late-onset progressive neurologic symptoms observed were identical to those previously described in adults with CHS with a mild clinical course of the disease and who did not undergo BMT^2^.

In addition to the clinical manifestations and laboratory examination, molecular studies are also essential for the diagnosis of this disease. Pathologic mutations in the lysosomal trafficking regulator gene (*LYST*, also known as Chediak-Higashi Syndrome1, *CHS1*) at 1q42.1–2 are responsible for this defect^6^,^7^. LYST is a member of the BEACH (beige and Chediak-Higashi) family, defined by the presence of a BEACH motif in the C-terminal. The BEACH motif consists of a WIDL-enriched sequence followed by several WD-40 domains known to serve as a platform for protein-protein interaction^8^,^9^,^10^. The N-terminal of LYST has several ARM/HEAT repeats known to play a role in membrane interaction^10^. To date, 41 mutations of *LYST* associated with Chediak-Higashi syndrome have been reported (Table 1)^11^. These mutations are located from nucleotide 118 to 11,173, predominantly after nucleotide 2,000 and largely (75.6%) leading to truncated mutants of LYST (Table 1).

In the present study, we report a new autosomal recessive form of Chediak-Higashi syndrome with atypical symptom, including partial oculocutaneous albinism, frequency upper respiratory infection or a marginal intelligence. Furthermore, WGS results showed chr1:235969126G > A (rs80338652)/chr1: 235915327A > AT compound heterozygote (R1104X/N2535KfsX2) in *LYST*. While LYST R1104X has been previously reported^7^, a novel LYST mutation N2535KfsX2 identified is expected to result in a deletion of the protein. These provide new laboratory evidence for the clinical and prenatal diagnosis of CHS.

## Results

### Clinical features

**Figure 1:**
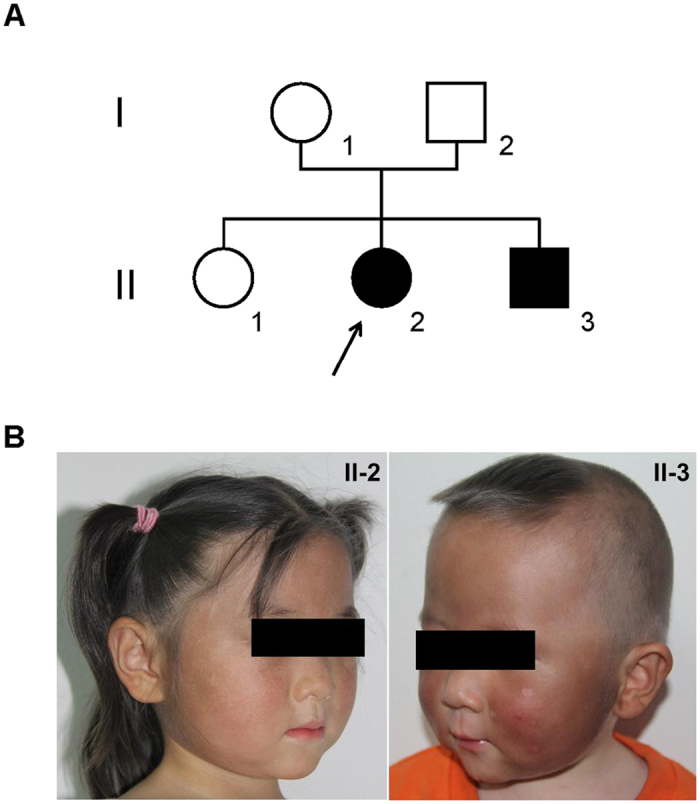
(**A**) Pedigree of the two-generation, Chinese family with two affected individuals. Squares indicate males, and circles represent females. Black and white symbols represent affected and unaffected individuals, respectively. The proband is indicated by an arrow. (**B**) Partial oculocutaneous albinism. Face of the proband(II-2) and younger brother(II-3). The two patients were noted that hyperpigmentation with bleaching spots of the cheeks and “silvery” colored hair.

**Figure 2:**
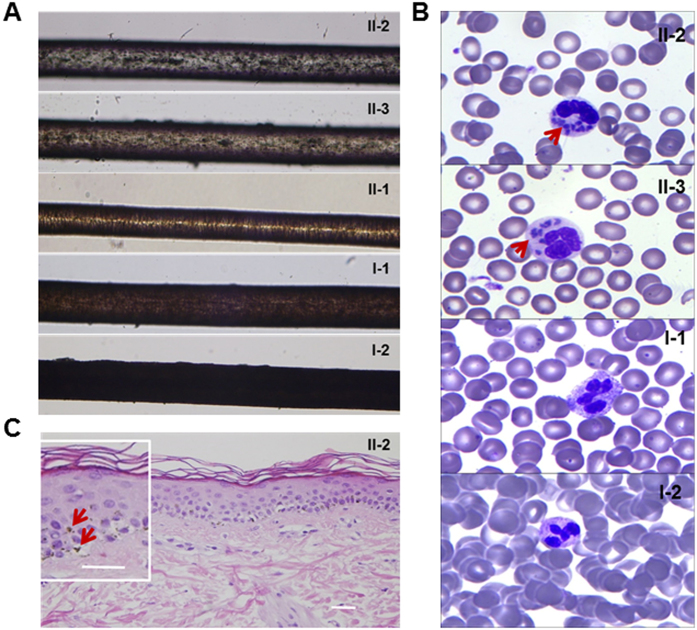
(**A**) Hair shafts. Irregular coarse melanin granules distributed in the patient’s hair (II-2 and II-3), evenly distributed pigment in the normal hair (II-1, I-1 and 1–2). (**B**) Wright staining of peripheral smear. Peripheral smear showed a polymorphonuclear leukocyte with abundant giant intracytoplasmic granules (100 ×) of the patients (II-2 and II-3 arrow), and normal polymorphonuclear leukocyte inclusion of unaffected members (I-1, I-2). (**C**) The proband left temporal skin biopsy (HE × 400). Skin projections becomes flat, basal cells are edematous, intracellular pigment particles are increasing, a small amount of lymphoid tissue cells exists in superficial dermal perivascular, occasionally pigment cells infiltration is visible (narrow).

The proband was a 6-year-old girl who presented to a dermatologist because of the appearance of darkening skin on her face (Fig. 1B left) and limbs (Supplementary Figure 1), noted “silvery” colored hair with an abnormal pigment clumping under light microscopy (Fig. 2A). A peripheral blood smear identified an abnormal WBC granules compared with unaffected members of the family (Fig. 2B). Left temporal skin biopsy of the proband (HE × 400) identified edema and an increased number of intracellular pigment particles in basal epithelial cells (Fig. 2C). The parents reported a normal pregnancy and delivery; however, in the first six months of life progressive hyperpigmentation of the cheeks and limbs extensor side, gray hair and mild photophobia appearance was noted. History revealed frequent upper respiratory infection of the proband, no family history of CHS, no history of severe re-infection and mild bleeding tendency. Physical examination revealed a height of 105 cm, 3% below the normal height of Chinese children. Fundus examination demonstrated abnormal retinal vascular and pigments (data not shown). Intriguingly, Wechsler Intelligence Scale test noted that the proband had a marginal intelligence, however cerebral MRI showed no obvious abnormality (Supplementary Figure 2).

The second case was the younger brother (1 year old) of the proband, with essentially the same clinical presentations. In this case, brown pigmentations are visible on the patient’s cheeks (Fig. 1B right). His hair was a“silvery” color (Fig. 1B right), and light microscopy examination revealed the presence of small, homogeneously distributed pigment clumps. These findings were in contrast to the normal pattern of distribution of unaffected family members (Fig. 2A). Peripheral blood smear showed giant inclusions in the cytoplasm of polymorphonuclear neutrophils (PMNs) not observed in the parents (Fig. 2B).

### Whole genome sequencing data analysis

Since the two affected siblings have a unique phenotype and no history of re-infection and bleeding, we performed whole genome sequencing of all family members to identify the causative variants. Average coverage for the affected and unaffected members was >60× and >30×, respectively (Table 2). To improve the reliability of variant calls, we retained variants identified by VarScan and GATK pipelines^12^,^13^ (see Methods). In total, we observed 5,246,911 positions at which at least one family member had an allele that varied from the reference genome. On average, each family member had approximately four million positions with variant, identified and annotated using ANNOVAR^14^ (MAF < 0.01 in Asian cohort by 1000 Genomes Project (2012 April) annotations, see Methods).

To search for possible causative variants in coding and potential regulatory regions, we hypothesized that the causative variants would be recessive, loss of heterozygosity (LOH) or compound heterozygous. 28 and 11 variants were identified by recessive and LOH model analyses. These variants distribute in coding, upstream (<1000 bp to TSS) or 5′ UTR regions as shown in Supplementary Table 1. Of the recessive variants, we observed only one missense variant of a chimeric POTE-actin protein (POTEF, chr1:130877752T > C), while the altered amino acid falls within nonfunctional region of POTEF protein. Although 25 recessive and 11 LOH variants located within upstream or 5′ UTR regions, there were no conserved transcriptional factor binding sites annotated by ANNOVAR. Thus, there were no obvious recessive and LOH variants in potential regulatory and coding regions that could be responsible for this disease. Besides, we identified four genes (LYST, ALMS1, TAS2R46, and TAS2R43) in which mutations identified are consistent with a compound heterozygous mode of inheritance (Supplementary Table 2). Two variants, chr1:235969126G > A (rs80338652) and chr1: 235915327A > AT, fall in the coding regions of LYST (Lysosomal Trafficking Regulator), and were predicted to be stop-gain and a frameshift insertion (Table 3), respectively. These two variants together comprise multiple alleles formed compound heterozygotes.

### Validation of mutations by Sanger sequencing

**Figure 3:**
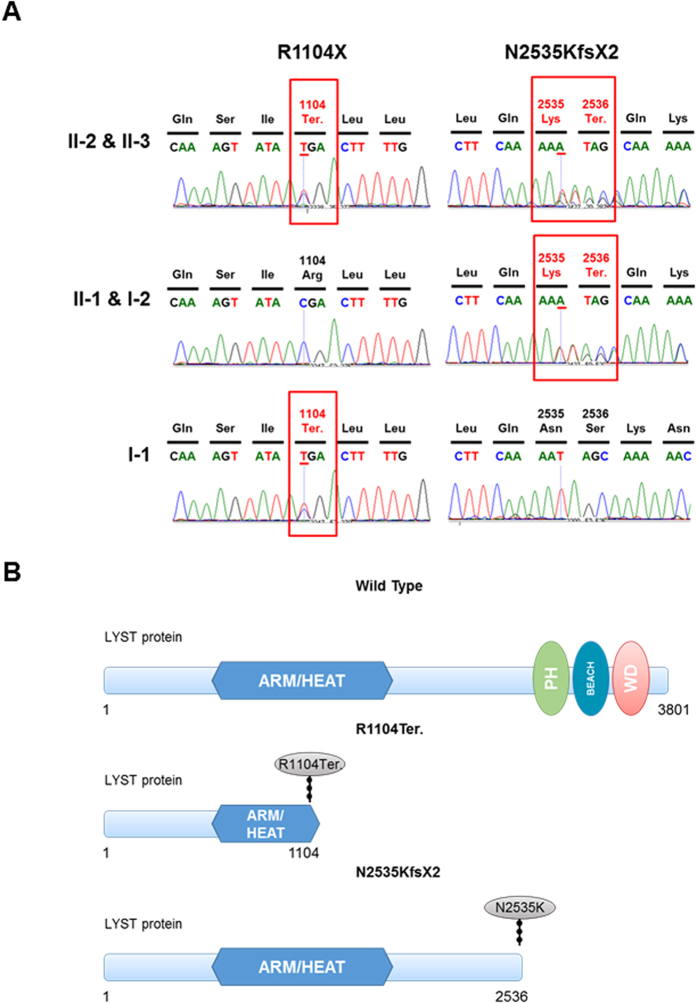
(**A**) Sanger sequencing validation studies. Sanger sequencing confirmed the compound heterozygotes mutations chr1:235969126G > A (rs80338652) (R1104Ter) and chr1: 235915327A > AT (N2535KfsX2) of the *LYST* gene identified. The two patients (II-2 and II-3) carry both of these two variants. The elder sister, who is unaffected, carries chr1: 235915327A > AT mutant. The father carries a chr1:235969126G > A (rs80338652) mutant, while mother carries chr1: 235915327A > AT mutant. (**B**) Human LYST protein and mutants. Domains and motifs responsible diagram of the mutations affected LYST protein. ARM/HEAT, the ARM/HEAT repeats region; PH, PH domain-like superfamily; BEACH, a BEACH motif in the C-terminal; WD, WD-40 domains. Recurrent R1104Ter mutationlocates in the ARM/HEAT repeats region. Mutation N2535KfsX2 leads to truncated of LYST protein at 2536 amino acid leading to deletion of PH-BEACH-WD domain.

We validated the two variants by Sanger sequencing (Fig. 3A). The screening sample included genomic DNA from frozen samples. Our results demonstrated that the father carries a chr1:235969126G > A (rs80338652) mutant, while mother carries a chr1: 235915327A > AT mutant. The affected patients carry both variants. The older sister (II-1), who is unaffected, carries a single LYST mutant (chr1: 235915327A > AT).

### Serotonin in the plasma of patients with CHS

**Figure 4:**
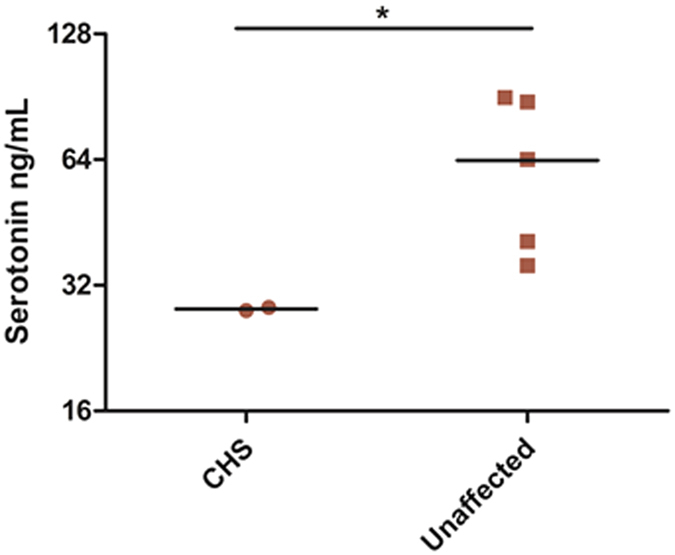
Serotonin levels in plasma of CHS subjects. Data are presented as medians. Plasma serotonin level is lower in CHS patients (II-2 and II-3) compared with healthy controls (Unaffects; II-1, I-1, I-2 and two healthy controls).

Absent or reduced number and irregular morphology of platelet-dense bodies is one of the characteristics in CHS patients. Due to serotonin is the main component of dense bodies, we assumed that CHS patients may have lower level of serotonin in the plasma. ELISA was performed to detect the plasma level of serotonin. Although unaffected members of the family are healthy, each individual carries chr1: 235969126G > A (rs80338652) or chr1: 235915327A > AT mutant. We thus add two unrelated healthy volunteers as control. The result shows that patients (II-2 and II-3, Serotonin 28.08 (27.83–28.32) ng/mL) with CHS exhibited significantly decreased plasma serotonin levels compared with unaffected family controls (II-1, I-1, I-2); plus two unrelated healthy controls (63.72 (35.66–90.17) ng/mL, *p* < 0.05* see Fig. 4).

## Discussion

The dermatologist diagnosed two patients in the family as atypical CHS with reiterative upper respiratory tract infection, without bleeding tendency, neurodegeneration and severe immunodeficiency. Previously these patients had been diagnosed with griscelli syndrome. We performed WGS analysis on five samples derived from the two patients, their sibling and parents, to characterize the underlying genetic mechanisms resulting in the atypical presentation. We identified a novel compound heterozygous mutations generating truncated LYST and leading to autosomal recessive CHS. Intriguingly, Wechsler Intelligence Scale Test unveiled the proband appears a marginal intelligence phenotype, thus excluding intelligence related mutations. The R1104X mutation was reported to lead to truncated LYST, causative of autosomal recessive CHS^7^. The novel mutation N2535KfsX2, results in a frameshift and consequent translational termination at codon 2536, disrupting important domains such as PH-BEACH-WD (Fig. 3B). Thus, this compound heterozygous mutations R1104X/N2535KfsX2 lead to autosomal recessive CHS. The present study is the first time to report the novel mutation chr1: 235915327A > AT, along with chr1:235969126G > A (rs80338652) together, forming a compound heterozygous in *LYST*. These provide new laboratory evidence for the clinical and prenatal diagnosis of CHS. Besides, our findings also reveal, for the first time, decreased plasma serotonin levels in patients with CHS compared with unaffected individuals.

In addition to the present study, previous study has reported another case carrying compound heterozygous for two *LYST* gene mutations, both of which are predicted to result in truncated proteins^1^. The two mutations are a nonsense mutation (c.1540 C > T, CGA > TGA, R514X) and a one base pair deletion (del c.9893 T, F3298fsX3304), coding for part of the LYST protein’s BEACH domain^1^. To date, including our study, two pairs of compound heterozygous *LYST* gene mutations have been found. Although CHS is known as a monogenic disease, next generation sequencing technology provides us a perfect tool to investigate potential gene mutations that may have effects on this rare disease. We performed whole genome sequencing to search for possible causative variants. We used recessive, loss of heterozygosity (LOH) or compound heterozygous modes to call rare mutations which have potential regulatory functions. The 28 recessive and 11 LOH variants were identified felling in coding, upstream or 5′ UTR regions. However, there were no obvious recessive and LOH variants in potential regulatory and coding regions that could be responsible for this disease. In compound heterozygous mode of inheritance, besides chr1:235969126G > A (rs80338652) and chr1: 235915327A > AT in LYST, one pair of compound heterozygous mutations in ALMS1, three pairs of compound heterozygous mutations in TAS2R43, and three pairs of compound heterozygous mutations in TAS2R46 were identified (see Supplementary Table 2). In ALMS1, F926S and P2960L compound heterozygous mutations falls within nonfunctional region, indicating these mutations don’t play key role CHS disease. In TAS2R43, S11I and R268G mutations fall in transmembrane-region 1 and transmembrane-region 7, respectively. Similarly, in TAS2R46, E265Q and T232I mutations fall in transmembrane-region 7 and transmembrane-region 6. TAS2R43 and TAS2R46 belong to the TAS2R family, and expressed on the surface of taste receptor cells, mediating the perception of bitterness through a G protein-coupled second messenger pathway^15^. Since the expression and function of TAS2R43 and TAS2R46 are specific related to taste, we considered these compound heterozygous mutations not related to CHS disease.

The diagnosis of CHS should be considered in individuals with pigment dilution defects of the hair, skin, or eyes; congenital or transient neutropenia; immunodeficiency; and otherwise unexplained neurologic abnormalities or neurodegeneration. The differential diagnosis of CHS with several diseases should be noted, such as oculocutaneous albinism (OCA), hermanskypudlak syndrome (HPS), griscelli syndrome (GS). Each of these diseases can be distinguished with CHS by the presence of specific molecular findings. OCA can be caused by *TYR, TYRP1* or *MATP* gene mutation; GS may be caused by pathogenic variants in *MYO5A, RAB27A* or *MLPH*; at least nine subtypes of hermansky-pudlak syndrome (HPS), HPS2 caused by variants in *AP3B1* which most closely resembles CHS. GS is characterized by mild skin hypopigmentation and silvery gray hair, but normal platelet function; these clinical features are highly similar to the phenotypic presentation of our patients. Although symptoms are similar among these diseases and CHS, the peripheral blood smear is sufficient to distinguish CHS. Moreover, *LYST* is the only gene known to be associated with CHS, thus genetic testing represents an important component to confirm the diagnosis^16^,^17^. In this study, the compound heterozygous mutations in *LYST* were shared by both patients. R1104X in LYST has been reported in CHS patients with decreased cognitive abilities^2^. However, in the present study, the R1104X heterozygote mutation carrier (mother of the patients) appears to have normal intelligence. Deletion of the PH-BEACH-WD domain of LYST can be caused by both R1104X and N2535KfsX2 mutations. We thus considered the compound heterozygous mutation in *LYST* as causative of the marginal intelligence in the proband. Interestingly, previous studies have shown that cerebral MRI findings consisted of supratentorial and cerebellar volume loss^2^. In the proband, however, cerebral MRI observed no obvious abnormalities. Several studies have been focused on intellectual limitation. Xue *et al*. found several genes (WNT1, BMP4, POU3F4, TFAP2C, PAX3, KCNA1, KCNC3, KCNG4, KCNJ3, KCNK9 and KCNT1) associated with neuronal differentiation of fragile X syndrome, which is a common hereditary disorder associated with intellectual limitations^18^. Besides, Posthuma *et al*. found ATXN1 and TRIM31 genes are related to intelligence in an attention deficit/hyperactivity disorder background^19^. However, we didn’t found any mutation fall in these genes. In order to expand our study, long-term follow-up observation of patients’ nervous system is necessary. Pigment dilution defects of the hair, skin, or eyes and large granules in leucocytes could support the diagnosis of CHS. The nature of CHS is due to various *LYST* gene mutations that produce different phenotypes^3^,^20^. Presently, clinicians and researches have been trying to further describe the relationship between genetic mutations and clinical symptoms. Oversized melanosomes accumulate in the perinuclear area are observed in epidermal melanocytes of individuals with CHS^3^. They are not transferred to surrounding keratinocytes, which would explain the skin hypomelanosis. Enlarged mature lytic granules in CHS cytotoxic T-lymphocytes are unable to mediate targeted cell killing^21^. Lack of platelet dense body can cause coagulation disorders^22^. Thus the specific role of LYST mutants requires further studies.

LYST is a member of the BEACH (beige and Chediak-Higashi) family of proteins, which are defined by the presence of a C-terminal BEACH motif. The BEACH motif consists of a WIDL-enriched sequence followed by several WD-40 domains known to serve as a platform for protein-protein interaction^8^,^9^,^10^. The N-terminal of LYST has several ARM/HEAT repeats known to play a role in membrane interaction^10^. Mutation chr1:235969126G > A (rs80338652) leads to truncated ARM/HEAT domain in sister chromatid, while chr1: 235915327A > AT results in deletion of PH-BEACH-WD domain in the other one. Thus the two mutations implement sister chromatid truncated LYST protein. The R1104X mutation (caused by rs80338652 G > A) in LYST is located in the ARM/HEAT repeats region, and result in LYST translational termination at codon 1104, indicating that may affect the membrane interaction function and protein-protein interaction of LYST (Fig. 3B). The N2535KfsX2 (caused by chr1: 235915327A > AT mutant) mutation is located between ARM/HEAT repeats region and PH domain and leads translational termination of LYST at codon 2536. Thus, we assumed that N2535KfsX2 mutant may not affect membrane interaction but affect protein-protein interaction.

A previous study identified twenty-one proteins that interact with LYST using a yeast two-hybrid screens^23^. R1104X mutation in LYST leads to loss of interaction with 18 proteins, that likely play key roles in vesicle tethering, docking and fusion, phosphorylation and signal transduction (Such as 14-3-3 proteins, Hepatocyte growth factor–regulated tyrosine kinase substrate and Casein kinase II β-subunit). Only 14-3-3β/τ and estrogen receptor-related protein (ERR1), involved in protein kinase C (PKC) signaling transduction, exocytosis and transcriptional regulation, can interact with R1104X truncation. LYST N2535KfsX2 truncation results in loss of 9 interaction proteins, involved in neurodegenerative disorder like Atrophin-1, transcription regulation and signal transduction. Thus, N2535KfsX2 can alleviate the symptoms caused by R1104X, thereby explaining the phenotype of our patients.

Several studies have been focused on varies of CHS phenotype by using LYST mutation mice. Two mouse strains, C57BL/6 souris mice and beige mice, are commonly used in CHS mechanism studies. Souris mice have a truncated mutation which ending at 2482 amino acid within the ARM/HEAT domain of LYST. Beige mice have an Ile deletion at 3741 amino acid within the WD domain. These two strains of mice have similar phenotype: diluted coat color, typical inclusion bodies in granulocytes and NK-cell degranulation and cytotoxicity^24^. Although both strains lacked NK-cell cytotoxicity, only souris mice developed all clinical and histopathologic signs of hemophagocytic lymphohistiocytosis (HLH) after infection with lymphocytic choriomeningitis virus^24^. In addition, CTLs in beige strain eliminated virus infection, while souris CTLs failed to control them. This suggests CTL cytotoxicity may be predictive for the risk of CHS patients to develop HLH^24^. Thus, we will follow up this family, investigating how LYST mutations affect CTLs function accelerating HLH in CHS for further study.

Most CHS platelets show absent dense bodies; a few platelets harbor enlarged and irregularly shaped dense bodies)^3^. Due to serotonin is the main component of dense bodies, we assumed that CHS patients may have lower level of serotonin in the plasma. As a result, our finding reveals decreased plasma serotonin levels in patients with CHS compared with unaffected individuals for the first time. Absences of dense bodies in CHS result in mild bleeding tendencies due to the inability to form hemostatic plugs, however, lower levels of serotonin in our patients don’t lead to bleeding tendency.

In the present study, we report a novel autosomal recessive form of CHS–R1104X/N2535KfsX2 compound heterozygote in LYST. R1104X recessive mutation in LYST has been previously reported^7^. Herein, we report a novel LYST mutation N2535KfsX2 together with R1104X is expected to result in a deletion protein, which leads to truncated ARM/HEAT domain and deletion of PH-BEACH-WD domain of LYST of the two sister chromatid respectively, in a newly diagnosed child and her family and provide phenotypic correlations via a thorough description of the affected individual. Thus this study contributes to improved understanding of the causes of this disease and provides new ideas for possible treatments.

## Methods

### Ethics Statement

The present study was approved by the Ethics Committee of the Beijing Children’s Hospital Affiliated to the Capital Medical University, Beijing, China, and was conducted according to the principles expressed in the Declaration of Helsinki. Participants and/or their legal guardians involved in this study gave a written informed consent prior to inclusion in the study.

### Patients

This study included two patients (II-2 and II-3) and three unaffected family members (elder sister (II-1), mother and father) as shown in Fig. 1A. Genomic DNA samples were obtained with written informed consent. TIAN amp Blood DNA Kit (Tiangen Biotech co., Ltd, Beijing) was used for extracting genomic DNA from blood samples.

### Reads mapping & variants calling

Paired-end reads of 300 bp (150 bp at each end) were mapped to UCSC human reference genome (GRCh37/hg19 assembly) using BWA^25^ version 0.7.7-r441 ‘mem’ mode with default options followed by removal of PCR duplicates, low-quality reads (BaseQ < 20). After being sorted and indexed by samtools^26^, the bam files of each offspring and the parents were converted as three-sample (trio) mpileup format. Variant calls were performed by VarScan software using version 2.3.7 of Trios pipeline^12^ (http://varscan.sourceforge.net/). In addition, the Genome Analysis Toolkit (GATK)^12^ pipeline with version 2.6–5 was used to cross-validate the variants called by VarScan.

### Variant annotation and prioritization

We used the ANNOVAR software for variant filtering and annotation^13^. Rare variants (minor allele frequency (MAF) <0.01 in Asian cohort) were filtered by 1000 Genomes Project data (2012 April) using ANNOVAR filter-based annotation^14^. By excluding common variants, the remaining rare variants were annotated by ANNOVAR RefSeq gene-based annotation^13^. In addition to removing synonymous, non-frameshift or noncoding variants, we also applied both recessive and compound heterozygous mode of inheritance to select candidate pathogenic variants.

### Data Availability

All the clinical data and identified genetic variations have been deposited into the rare disease database - eRAM at http://www.unimd.org/eram/.

### Amplification mutation sites and Sanger sequencing

We amplified the two mutations in *LYST* with a Veriti 96-well Thermal Cycler (Applied Biosystems, Thermo Fisher Scientific). The primer sequences used include: F: 5′-cag aagtcacaaagaggagca-3′, R: 5′-ttgtgtgatactgttggctgc-3′; F: 5′-tacatattacttgccatagaa-3′, R: 5′-agttggcaatacataagtga-3′. Mutations were confirmed by Sanger sequencing.

### Enzyme-Linked Immunosorbent Assay (ELISA)

The plasma level of serotonin was measured by ELISA using a commercial serotonin antibody kit according to the manufacturer’s instructions (ab133053; Abcam, Cambridge, MA, USA). All ELISA measurements were made in duplicate and results were expressed as median values and interquartile ranges. Comparisons between two groups were made using a two-tailed Mann-Whitney U-test. A *p*-value of 0.05 was considered significant.

## Additional Information

**How to cite this article:** Jin, Y. *et al*. Whole Genome Sequencing Identifies Novel Compound Heterozygous Lysosomal Trafficking Regulator Gene Mutations Associated with Autosomal Recessive Chediak-Higashi Syndrome. *Sci. Rep.* **7**, 41308; doi: 10.1038/srep41308 (2017).

**Publisher's note:** Springer Nature remains neutral with regard to jurisdictional claims in published maps and institutional affiliations.

## Supplementary Material

Supplementary Files

## Figures and Tables

**Table 1:** LYST Pathogenic Allelic Variants.

NO.	DNA Change	Amino Acid Change	NO.	DNA Change	Amino Acid Change	NO.	DNA Change	Amino Acid Change	NO.	DNA Change	Amino Acid Change
1	c.118_119insG	p.Ala40GlyfsTer24	12	c.3085C > T	p.Gln1029Ter	23	c.5506C > T	p.Arg1836Ter	34	c.9228_9229ins TTCTTTCAGT	p.Lys3077PhefsTer4
2	c.148C > T	p.Arg50Ter	13	c.3310C > T	p.Arg1104Ter	24	c.5541_5542delAA	p.Gln1847fsTer1850	35	c.9590delA	p.Tyr3197LeufsTer62
3	c.925C > T	p.Arg309Ter	14	c.3434dupA	p.His1145GlnfsTer9	25	c.5996T > A	p.Val1999Asp	36	c.9827_9832ATACAA	p.Asn3276_Thr3277del
4	c.772T > C	p.Cys258Arg	15	c.3622C > T	p.Gln1208Ter	26	c.6078C > A	p.Tyr2026Ter	37	c.9893delT	p.Phe3298Serfs7
5	c.1467delG	p.Glu489AspfsTer78	16	c.3944_3945 insC	p.Thr1315fsTer1331	27	c.7060_7066del CTATTAG	p.Leu2354 MetfsTer16	38	c.10127A > G	p.Asn3376Ser
6	c.1540C > T	p.Arg514Ter	17	c.4052C > G	p.Ser1351Ter	28	c.7555delT	p.Tyr2519 IlefsTer10	39	c.10395delA	p.Gly3466AlafsTer2
7	c.1902dupA	p.Ala635SerfsTer4	18	c.4361 C > A	p.Ala1454Asp	29	c.7982C > G	p.Ser2661Ter	40	c.11102G > T	p.Glu3668Ter
8	c.2413delG	p.Glu805AsnfsTer2	19	c.4274delT	p.Leu1425TyrfsTer2	30	c.8281A > T	p.Arg2761Ter	41	c.11173G > A	p.Gly3725Arg
9	c.2454delA	p.Ala819Hisfs5	20	c.4688G > A	p.Arg1563His	31	c.8428G > A	p.Glu2810Lys			
10	c.2623delT	p.Tyr875MetfsTer24	21	c.5061T > A	p.Tyr1687Ter	32	c.8583G > A	p.Trp2861Ter			
11	c.3073_3074 delAA	p.Asn1025GlnfsTer6	22	c.5317delA	p.Arg1773 AspfsTer13	33	c.9107_9162del56	p.Gly3036 GlufsTer16			

**Table 2:** Whole genome sequencing statistics for the family.

Family member	#of reads	#of variants	#rare variants (MAF <0.01)	Average coverage
II-2	1,294,717,932	3,884,626	1,019,066	64.74
II-3	604,579,332	3,643,750	921,560	30.23
II-1	1,271,485,728	3,850,633	1,017,205	63.57
I-2	706,084,970	3,819,074	982,427	35.30
I-1	650,834,656	3,826,470	996,484	32.54

MAF, minor allele frequency.

**Table 3:** Summary of two putative causative variants in LYST.

Effect	Gene	mRNA	exon	II-2	II-3	II-1	I-2	I-1
frameshift insertion	LYST	NM_000081	exon27:c.7605_7605delinsAT	het.	het.	het.	het.	wild
stopgain SNV	LYST	NM_000081	exon6:c.C3310T:p.R1104X	het.	het.	wild	wild	het.

Het., heterozygous genotype; wild, wild type.

## References

[b1] Zarzour W. . Two novel CHS1 (LYST) mutations: clinical correlations in an infant with Chediak-Higashi syndrome. Molecular genetics and metabolism 85, 125–132, doi: 10.1016/j.ymgme.2005.02.011 (2005). 15896657

[b2] Tardieu M. . Progressive neurologic dysfunctions 20 years after allogeneic bone marrow transplantation for Chediak-Higashi syndrome. Blood 106, 40–42, doi: 10.1182/blood-2005-01-0319 (2005). 15790783

[b3] Westbroek W. . Cellular defects in Chediak-Higashi syndrome correlate with the molecular genotype and clinical phenotype. J Invest Dermatol 127, 2674–2677 (2007). 17554367 10.1038/sj.jid.5700899

[b4] Faigle W. . Deficient peptide loading and MHC class II endosomal sorting in a human genetic immunodeficiency disease: the Chediak-Higashi syndrome. J Cell Biol 141, 1121–1134 (1998). 9606205 10.1083/jcb.141.5.1121 PMC2137185

[b5] Ambrosio A. . Mechanism of platelet dense granule biogenesis: study of cargo transport and function of Rab32 and Rab38 in a model system. Blood 120, 4072–4081 (2012). 22927249 10.1182/blood-2012-04-420745 PMC3496959

[b6] Barbosa M. D. . Identification of the homologous beige and Chediak-Higashi syndrome genes. Nature 382, 262–265 (1996). 8717042 10.1038/382262a0 PMC2893578

[b7] Nagle D. L. . Identification and mutation analysis of the complete gene for Chediak-Higashi syndrome. Nat Genet 14, 307–311 (1996). 8896560 10.1038/ng1196-307

[b8] Jogl G. . Crystal structure of the BEACH domain reveals an unusual fold and extensive association with a novel PH domain. EMBO J 21, 4785–4795 (2002). 12234919 10.1093/emboj/cdf502 PMC126298

[b9] De Lozanne A. The role of BEACH proteins in Dictyostelium. Traffic 4, 6–12 (2003). 12535270 10.1034/j.1600-0854.2003.40102.x

[b10] Kaplan J., De Domenico I. & Ward D. M. Chediak-Higashi syndrome. Curr Opin Hematol 15, 22–29 (2008). 18043242 10.1097/MOH.0b013e3282f2bcce

[b11] Karim M. A. . Apparent genotype-phenotype correlation in childhood, adolescent, and adult Chediak-Higashi syndrome. Am J Med Genet 108, 16–22 (2002). 11857544

[b12] Koboldt D. C. . VarScan 2: somatic mutation and copy number alteration discovery in cancer by exome sequencing. Genome Res 22, 568–576 (2012). 22300766 10.1101/gr.129684.111 PMC3290792

[b13] Van der Auwera G. A. . From FastQ data to high confidence variant calls: the Genome Analysis Toolkit best practices pipeline. Curr Protoc Bioinformatics 11, 11.10.11–11.10.33 (2013). 10.1002/0471250953.bi1110s43 PMC4243306 25431634

[b14] Wang K., Li M. & Hakonarson H. ANNOVAR: functional annotation of genetic variants from high-throughput sequencing data. Nucleic Acids Res 38, e164 (2010). 20601685 10.1093/nar/gkq603 PMC2938201

[b15] Conte C. . Identification and characterization of human taste receptor genes belonging to the TAS2R family. Cytogenet Genome Research 98(1), 45–53 (2002). 10.1159/000068546 12584440

[b16] Introne W. J. . Chediak-Higashi Syndrome in GeneReviews® (ed. Pagon R. A. .) (Seattle, 1993–2016). 20301751

[b17] Huizing M. . A. Disorders of lysosome-related organelle biogenesis: clinical and molecular genetics. Annu Rev Genomics Hum Genet 9, 359–386 (2008). 18544035 10.1146/annurev.genom.9.081307.164303 PMC2755194

[b18] Lu P. . Integrated transcriptome analysis of human ips cells derived from a fragile × syndrome patient during neuronal differentiation. Science China Life sciences (2016). 10.1007/s11427-016-0194-6 27730449

[b19] Rizzi T. S. . The atxn1 and trim31 genes are related to intelligence in an adhd background: Evidence from a large collaborative study totaling 4,963 subjects. American journal of medical genetics Part B, Neuropsychiatric genetics: the official publication of the International Society of Psychiatric Genetics 156, 145–157 (2011). 10.1002/ajmg.b.31149 PMC3085124 21302343

[b20] Kaya Z. . A novel single point mutation of the LYST gene in two siblings with different phenotypic features of Chediak Higashi syndrome. Pediatr Blood Cancer 56, 1136–1139 (2011). 21488161 10.1002/pbc.22878

[b21] Holland P. . LYST affects lysosome size and quantity, but not trafficking or degradation through autophagy or endocytosis. Traffic 15, 1390–1405 (2014). 25216107 10.1111/tra.12227

[b22] Golebiewska E. M. & Poole A. W. Platelet secretion: From haemostasis to wound healing and beyond. Blood Rev 29, 153–162 (2015). 25468720 10.1016/j.blre.2014.10.003 PMC4452143

[b23] Tchernev V. T. . The Chediak-Higashi protein interacts with SNARE complex and signal transduction proteins. Molecular medicine 8, 56–64 (2002). 11984006 PMC2039936

[b24] Jessen B. . Subtle differences in CTL cytotoxicity determine susceptibility to hemophagocytic lymphohistiocytosis in mice and humans with Chediak-Higashi syndrome. Blood 118(17), 4620–9, doi: 10.1182/blood-2011-05-356113 (2011). 21878672

[b25] Li H. Exploring single-sample SNP and INDEL calling with whole-genome de novo assembly. Bioinformatics 28, 1838–1844 (2012). 22569178 10.1093/bioinformatics/bts280 PMC3389770

[b26] Li H. . The Sequence Alignment/Map format and SAMtools. Bioinformatics 25, 2078–2079 (2009). 19505943 10.1093/bioinformatics/btp352 PMC2723002

