# Supplementary material for: Whole Genome Sequencing Identifies Novel Compound Heterozygous Lysosomal Trafficking Regulator Gene Mutations Associated with Autosomal Recessive Chediak-Higashi Syndrome
Source: Sci Rep. 2017 Feb 1;7:41308. doi: 10.1038/srep41308 (PMC5286514; doi:10.1038/srep41308)
Supplement: Supplementary Files [file srep41308-s1.pdf]

Whole Genome Sequencing Identifies Novel Compound Heterozygous Lysosomal Trafficking Regulator Gene Mutations Associated with Autosomal Recessive Chediak-Higashi Syndrome

Yaqiong Jin[1,2, #]; Li Zhang[3, #]; Senfen Wang[4]; Feng Chen[5]; Yang Gu[4]; Enyu Hong[2]; Yongbo Yu[1]; Xin Ni[1,2,5,]; Yongli Guo[1, 2, *]; Tieliu Shi[3, *];Zigang Xu[4, *]

[1]Beijing Key Laboratory for Pediatric Diseases of Otolaryngology, Head and Neck Surgery, MOE Key Laboratory of Major Diseases in Children, Beijing Pediatric Research Institute, Beijing Children's Hospital, Capital Medical University, Beijing, China

[2]Biobank for Clinical Data and Samples in Pediatric, Beijing Pediatric Research Institute, Beijing Children's Hospital, Capital Medical University, Beijing, China

[3]Center for Bioinformatics and Computational Biology, and the Institute of Biomedical Sciences, School of Life Sciences, East China Normal University, Shanghai, China

[4]Department of Dermatology, Beijing Children's Hospital, Capital Medical University, Beijing, China

[5]Department of Otolaryngology, Head and Neck Surgery, Beijing Children's Hospital, Capital Medical University, Beijing,China

[#]These authors contributed equally

***Corresponding author**

Zigang Xu

Department of Dermatology, Beijing Children'sHospital, Capital Medical University, 56 Nanlishi Rd. Beijing, 100045, China

E-mail zigangxu@yahoo.com (Zigang Xu).

Tieliu Shi

Center for Bioinformatics and Computational Biology, and the Institute of Biomedical Sciences, School of Life Sciences, East China Normal University, 500 Dongchuan Road, Shanghai, 200241, China

E-mail tieliushi@yahoo.com (Tieliu Shi).

Yongli Guo

Beijing Key Laboratory for Pediatric Diseases of Otolaryngology, Head and Neck Surgery, Beijing Pediatric Research Institute, Beijing Children's Hospital, Capital Medical University, 56 Nanlishi Rd. Beijing, 100045, China Fax: +1 870 543 7773.

E-mail guoyongli@bch.com.cn (YongliGuo).

Supplementary Figure 1

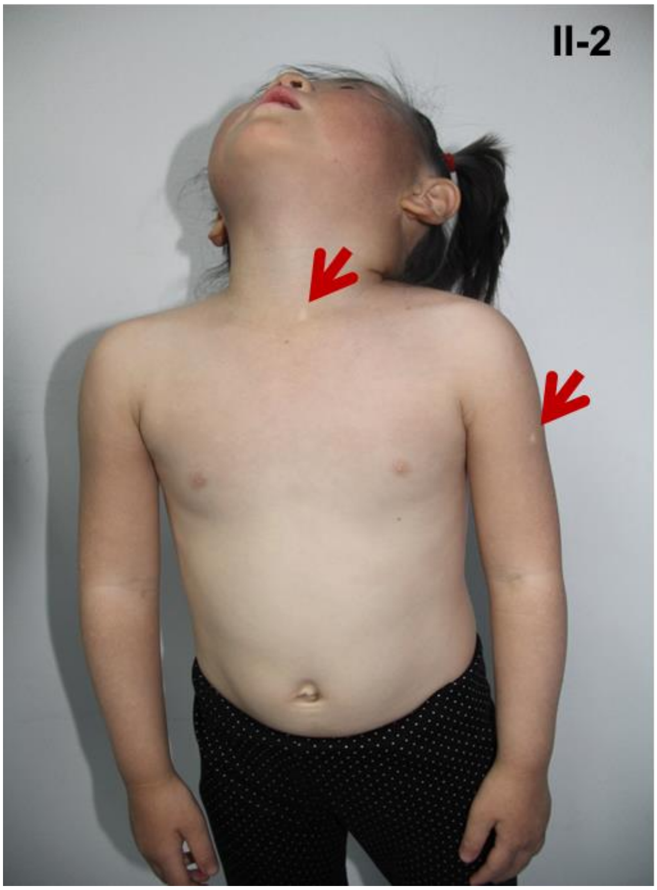

Supplementary Figure 2

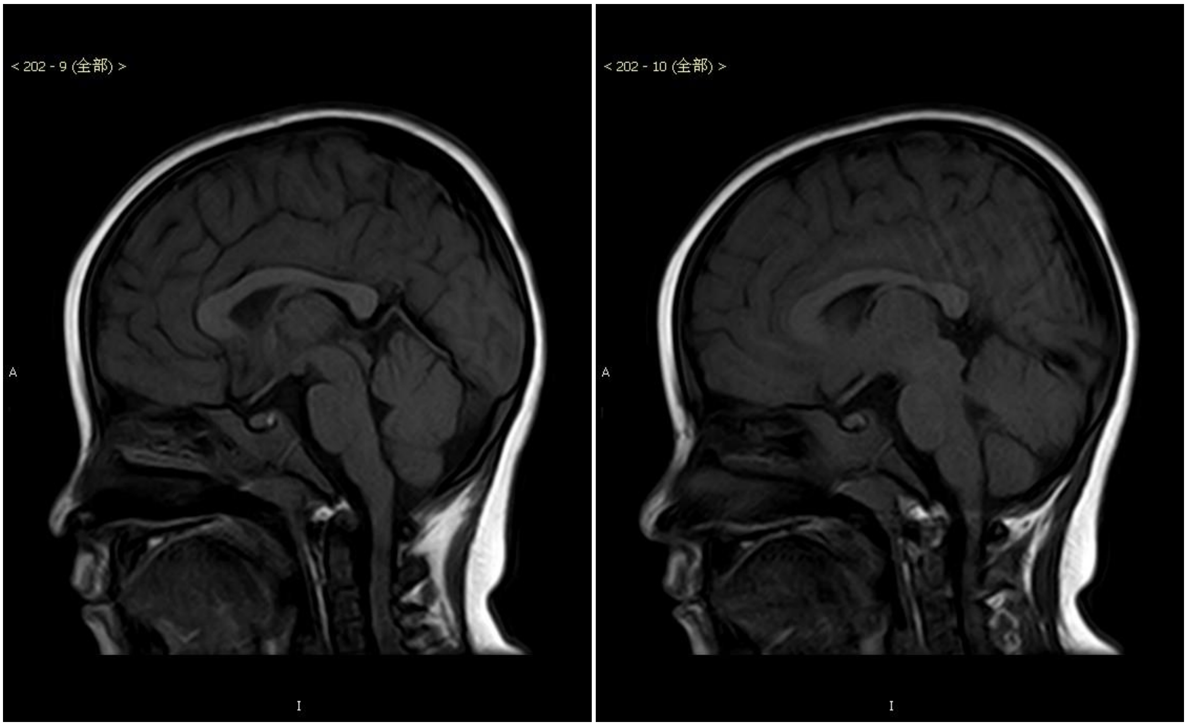

Supplementary Table S1 Summary of putative causative variants

Chr	Start	End	Ref	Alt	Effect	Region	Gene	Pattern	I-1	I-2	II-1	II-2	II-3
chr6	168398188	168398188	G	-	NO	upstream	KIF25-AS1	LOH	wild	het.	wild	hom.	hom.
chr4	57332913	57332913	A	T	NO	upstream	SRP72	LOH	wild	het.	het.	hom.	hom.
chr14	93799587	93799587	-	CGGGCGTCGGGTCGCTGGGAGT	NO	UTR5	UNC79	LOH	wild	het.	wild	hom.	hom.
chr11	65488470	65488486	CCCCTCTCCTTCCCCCT	-	NO	upstream	RNASEH2C	LOH	wild	het.	wild	hom.	hom.
chr2	242640727	242640727	T	G	NO	upstream	ING5	LOH	wild	het.	wild	hom.	hom.
chr14	51563010	51563010	-	G	NO	upstream	TRIM9	LOH	wild	het.	het.	hom.	hom.
chr1	40157517	40157517	-	CA	NO	upstream	HPCAL4	LOH	wild	het.	wild	hom.	hom.
chr3	172468193	172468193	-	A	NO	upstream	ECT2	LOH	het.	wild	wild	hom.	hom.
chr2	133064709	133064709	-	TTT	NO	upstream	ZNF806	LOH	het.	wild	het.	hom.	hom.
chr15	22146377	22146378	GT	-	NO	upstream	NF1P2	LOH	het.	wild	wild	hom.	hom.
chr6	44310168	44310168	-	A	NO	upstream	SPATS1	LOH	het.	wild	wild	hom.	hom.
chr2	178129391	178129393	GGC	-	NO	UTR5	NFE2L2	recessive	het.	het.	wild	hom.	hom.
chr2	237416264	237416264	C	A	NO	upstream	IQCA1	recessive	het.	het.	het.	hom.	hom.
chr3	125655833	125655833	A	G	NO	UTR5	ALG1L	recessive	het.	het.	wild	hom.	hom.

Chr	Start	End	Ref	Alt	Effect	Region	Gene	Pattern	I-1	I-2	II-1	II-2	II-3
chr11	75380407	75380407	G	C	NO	upstream	MAP6	recessive	het.	het.	wild	hom.	hom.
chr2	38103204	38103204	A	-	NO	upstream	LINC00211	recessive	het.	het.	het.	hom.	hom.
chr2	38892451	38892451	-	TTG	NO	upstream	GALM	recessive	het.	het.	wild	hom.	hom.
chr5	56866066	56866066	-	CTGTG TG	NO	upstream	LINCR-000 3	recessive	het.	het.	het.	hom.	hom.
chr22	25843612	25843612	G	A	NO	upstream	CRYBB2P 1	recessive	het.	het.	het.	hom.	hom.
chr4	6474241	6474241	G	C	NO	UTR5	PPP2R2C	recessive	het.	het.	het.	hom.	hom.
chr2	130832531	130832531	A	G	synonym ous	exonic	POTEF	recessive	het.	het.	het.	hom.	hom.
chr3	69435725	69435725	A	-	NO	upstream	FRMD4B	recessive	het.	het.	wild	hom.	hom.
chr11	94883646	94883646	T	C	NO	upstream	LOC10192 9295	recessive	het.	het.	wild	hom.	hom.
chr6	49467613	49467613	T	-	NO	upstream	GLYATL3	recessive	het.	het.	wild	hom.	hom.
chr3	132035970	132035970	A	-	NO	upstream	ACPP	recessive	het.	het.	wild	hom.	hom.
chr2	237416259	237416259	C	G	NO	upstream	IQCA1	recessive	het.	het.	het.	hom.	hom.
chr14	56046972	56046977	GCG GCG	-	NO	UTR5	KTN1	recessive	het.	het.	wild	hom.	hom.
chr22	25843608	25843608	A	G	NO	upstream	CRYBB2P 1	recessive	het.	het.	het.	hom.	hom.
chr5	53750939	53750939	-	TTTG	NO	upstream	HSPB3	recessive	het.	het.	wild	hom.	hom.
chr1	43735695	43735695	T	G	NO	UTR5	TMEM125	recessive	het.	het.	wild	hom.	hom.
chr2	132202667	132202667	T	C	NO	upstream	NOC2LP2	recessive	het.	het.	het.	hom.	hom.
chr11	67277153	67277153	-	C	NO	upstream	CDK2AP2	recessive	het.	het.	het.	hom.	hom.

**Chr**	**Start**	**End**	**Ref**	**Alt**	**Effect**	**Region**	**Gene**	**Pattern**	**I-1**	**I-2**	**II-1**	**II-2**	**II-3**
chr2	130877752	130877752	T	C	nonsynonymous	exonic	POTEF	recessive	het.	het.	het.	hom.	hom.
chr4	132685658	132685658	T	A	NO	upstream	LOC101927305	recessive	het.	het.	wild	hom.	hom.
chr22	50971639	50971639	-	A	NO	upstream	ODF3B	recessive	het.	het.	het.	hom.	hom.
chr5	56248735	56248735	C	A	NO	upstream	MIER3	recessive	het.	het.	wild	hom.	hom.
chr1	55505552	55505552	-	CTG	nonframe shift insertion	exonic	PCSK9	recessive	het.	het.	het.	hom.	hom.
chr2	130808939	130808939	A	G	NO	upstream	FAR2P1	recessive	het.	het.	het.	hom.	hom.
chr22	25843590	25843593	ACAC	-	NO	upstream	CRYBB2P1	recessive	het.	het.	wild	hom.	hom.

wild, wild type;het., heterozygous genotype; hom., homozygous genotype

Supplementary Table S2 Summary of two putative causative variants in LYST

Effect	Gene	mRNA	exon	II-2	II-3	II-1	I-2	I-1
frameshift insertion	LYST	NM_000081	exon27:c.7605_7605delinsAT	het.	het.	het.	het.	wild
stopgain SNV	LYST	NM_000081	exon6:c.C3310T:p.R1104X	het.	het.	het.	wild	het.
nonsynonymous SNV	ALMS1	NM_015120	exon8:c.T2777C:p.F926S	het.	het.	wild	wild	het.
nonsynonymous SNV	ALMS1	NM_015120	exon10:c.C8879T:p.P2960L	het.	het.	wild	het.	wild
nonsynonymous SNV	TAS2R43	NM_176884	exon1:c.G32T:p.S11I	het.	het.	wild	wild	het.
nonsynonymous SNV	TAS2R43	NM_176884	exon1:c.G893C:p.R298T	het.	het.	wild	het.	wild
nonsynonymous SNV	TAS2R43	NM_176884	exon1:c.A802G:p.R268G	het.	het.	het.	het.	wild
nonsynonymous SNV	TAS2R43	NM_176884	exon1:c.T882G:p.F294L	het.	het.	wild	het.	wild
nonsynonymous SNV	TAS2R46	NM_176887	exon1:c.G793C:p.E265Q	het.	het.	wild	wild	het.
nonsynonymous SNV	TAS2R46	NM_176887	exon1:c.C695T:p.T232I	het.	het.	wild	het.	wild
nonsynonymous SNV	TAS2R46	NM_176887	exon1:c.G757A:p.E253K	het.	het.	wild	het.	wild
nonsynonymous SNV	TAS2R46	NM_176887	exon1:c.C680T:p.A227V	het.	het.	wild	het.	wild

het., heterozygous genotype; wild, wild type.
